# Application of vibration to wrist and hand skin affects fingertip tactile sensation

**DOI:** 10.14814/phy2.12465

**Published:** 2015-07-14

**Authors:** Kishor Lakshminarayanan, Abigail W Lauer, Viswanathan Ramakrishnan, John G Webster, Na Jin Seo

**Affiliations:** 1 Department of Industrial and Manufacturing Engineering, University of Wisconsin-MilwaukeeMilwaukee, Wisconsin, USA; 2 Department of Public Health Sciences, Medical University of South CarolinaCharleston, South Caroline, USA; 3 Department of Biomedical Engineering, University of Wisconsin-MadisonMadison, Wisconsin, USA; 4 Division of Occupational Therapy, Department of Health Professions, Department of Health Sciences and Research, Medical University of South CarolinaCharleston, South Carolina, USA

**Keywords:** Finger, hand function, stochastic resonance, tactile sensation, vibration

## Abstract

A recent study showed that fingertip pads’ tactile sensation can improve by applying imperceptible white-noise vibration to the skin at the wrist or dorsum of the hand in stroke patients. This study further examined this behavior by investigating the effect of both imperceptible and perceptible white-noise vibration applied to different locations within the distal upper extremity on the fingertip pads’ tactile sensation in healthy adults. In 12 healthy adults, white-noise vibration was applied to one of four locations (dorsum hand by the second knuckle, thenar and hypothenar areas, and volar wrist) at one of four intensities (zero, 60%, 80%, and 120% of the sensory threshold for each vibration location), while the fingertip sensation, the smallest vibratory signal that could be perceived on the thumb and index fingertip pads, was assessed. Vibration intensities significantly affected the fingertip sensation (*P *< 0.01) in a similar manner for all four vibration locations. Specifically, vibration at 60% of the sensory threshold improved the thumb and index fingertip tactile sensation (*P* < 0.01), while vibration at 120% of the sensory threshold degraded the thumb and index fingertip tactile sensation (*P *<* *0.01) and the 80% vibration did not significantly change the fingertip sensation (*P *>* *0.01), all compared with the zero vibration condition. This effect with vibration intensity conforms to the stochastic resonance behavior. Nonspecificity to the vibration location suggests the white-noise vibration affects higher level neuronal processing for fingertip sensing. Further studies are needed to elucidate the neural pathways for distal upper extremity vibration to impact fingertip pad tactile sensation.

## Introduction

The objective of this study was to investigate the way the fingertip pads’ tactile sensation is affected by white-noise vibration applied to the distal upper extremity in healthy adults. Specifically, the effects of both imperceptible and perceptible white-noise vibration intensities applied at one of four locations (dorsum hand by the second knuckle, thenar and hypothenar areas, and volar wrist) were examined to improve understanding of its influence on fingertip pad tactile sensation.

Finger tactile sensation is a prerequisite for dexterous hand function including fine finger movements, gripping, and object manipulation (Zatsiorsky and Latash 2004). Anesthesia of the fingers results in immediate decline in grip strength, increase in safety margin, and slippage of an object from the hand in healthy adults (Johansson and Westling 1984; Augurelle et al. 2003; Monzee et al. 2003). Likewise, deficits in tactile sensation, such as following peripheral nerve injuries, aging (Stevens and Patterson 1995; Leveque et al. 2000; Kalisch et al. 2009), or stroke (Carey 1995; Turton and Butler 2001; Carey and Matyas 2011), can reduce sensory feedback from the fingers, resulting in inappropriate grip force control (Blennerhassett et al. 2006, 2007), deteriorated manual dexterity and fine object manipulation (Dannenbaum and Jones 1993; Tremblay et al. 2003), unstable grip (Seo et al. 2010), and dropping of objects (Pazzaglia et al. 2010).

Given the direct connection between finger tactile sensation and hand function, it is important to know potential sources that affect tactile sensation. Sensory manipulation exploiting these sources could be used to facilitate or degrade hand dexterity and hand functions depending on the application. One of the sensory manipulation techniques involves imperceptible vibration. Application of imperceptible vibration to the fingertips has been shown to improve the fingertip pad’s tactile sensation and reduce excessive grip force during object lifting (Kurita et al. 2013). Such a wearable vibrating device can be realized using a low-cost, low-risk mechanical vibrator, with an instant effect (Kurita et al. 2013) to enhance human performance in high-precision manual dexterity tasks, such as assembling intricate parts, playing music, or sports, and performing surgical procedures.

This vibration is thought to work based on a concept from traditional control theory in which presence of low-level random noise increases the signal to noise ratio and the system’s ability to respond to signals. Such a phenomenon is also referred to as “stochastic resonance” (Moss et al. 2004). In the human tactile sensory system, application of imperceptible white-noise vibration to the tactile signal resulted in improved detection of the tactile signal for the fingertip (Collins et al. 1997; Liu et al. 2002) as well as foot sole (Wells et al. 2005). This effect on sensation was supported by electrophysiological data showing that white-noise vibration resulted in increased signal to noise ratios in EEG somatosensory evoked responses (Manjarrez et al. 2002). Furthermore, there appears to be an optimal level of white-noise vibration for improving human tactile sensation: Wells et al. (2005) showed an inverted U-shaped relationship between noise intensity and sensation in which white-noise vibration at the intensities of 33%, 50%, and 67% of the sensory threshold improved tactile sensation to a greater extent than did vibration at intensities of 83% and 100% of the sensory threshold compared with baseline sensation with no vibration in healthy adults. White-noise vibration above sensory threshold was shown to degrade sensation, likely by masking the tactile signal and interfering with signal detection (Collins et al. 1997). Based on these results, Wells et al. (2005) concluded that white-noise vibration should be high enough to facilitate a weak tactile signal to cross a sensory threshold but not too high to mask the tactile signal.

Recent studies show that application of imperceptible white-noise vibration away from the fingertips such as the wrist or the dorsum of the hand may also improve fingertip tactile sensation: Imperceptible white-noise vibration applied to the dorsal and volar wrist as well as the dorsum of the hand by the first and second knuckles was found to improve the index and thumb fingertip pads’ light touch sensation in chronic stroke survivors (Enders et al. 2013). Imperceptible white-noise vibration applied to the thenar eminence and the volar and dorsal forearm skin shortened muscle reaction time to hand tactile stimuli in healthy adults, as did white-noise vibration applied to the middle fingertip (Hur et al. 2014).

Such a remote effect offers a practical benefit by strategically placing a vibrator off the hand in order to expose the entire hand skin for tactile stimuli during dexterous manual tasks and also to not interfere with object manipulation. In addition, this remote effect has the potential to expand our current understanding of sensory manipulation in the following way. We often assume that small vibratory noise on the base of the palm or wrist from laying the hand on a table or a wristband-type device would not affect finger sensation and dexterity. This assumption will be greatly challenged if we find that small vibratory noise around the palm or wrist effectively changes fingertip tactile sensation. Currently, it is unknown if imperceptible white-noise vibration applied to the upper extremity other than the fingertips can affect fingertip tactile sensation in healthy adults. In addition, it is currently unclear how this practical benefit of the remote effect is accrued.

This study aimed to investigate further the effect of white-noise vibration applied to the upper extremity other than the fingertips on fingertip tactile sensation in healthy adults by varying the noise locations and intensities. Specifically, to examine if connections between specific nerves are mediating the remote effect, we tested four vibration locations of the thenar eminence, hypothenar region, volar wrist, and dorsum of the hand just proximal to the second knuckle that are innervated by the median nerve, ulnar nerve, lateral musculocutaneous nerve, and radial nerve, respectively. The greatest effect of the vibration when applied to the thenar eminence compared to other locations may indicate involvement of median nerve sharing, whereas equal extents of sensory effects for all vibration locations may indicate involvement of higher level neural connections. In addition, three different noise intensities of 60%, 80%, and 120% of the sensory threshold were tested to examine if the remote vibration affects the fingertip tactile sensation in a manner similar to stochastic resonance.

## Methods

### Subjects

Twelve healthy right-handed adults (four females and eight males) with a mean age of 29 ± 5 ranging from 20 to 40 years participated in the study. All subjects verbally disclosed that they had no history of upper limb injury or musculoskeletal or neurologic disorders. The protocol was approved by the Institutional Review Board. Subjects read and signed a written informed consent form before participating in the experiment.

### Procedure

**Figure 1:**
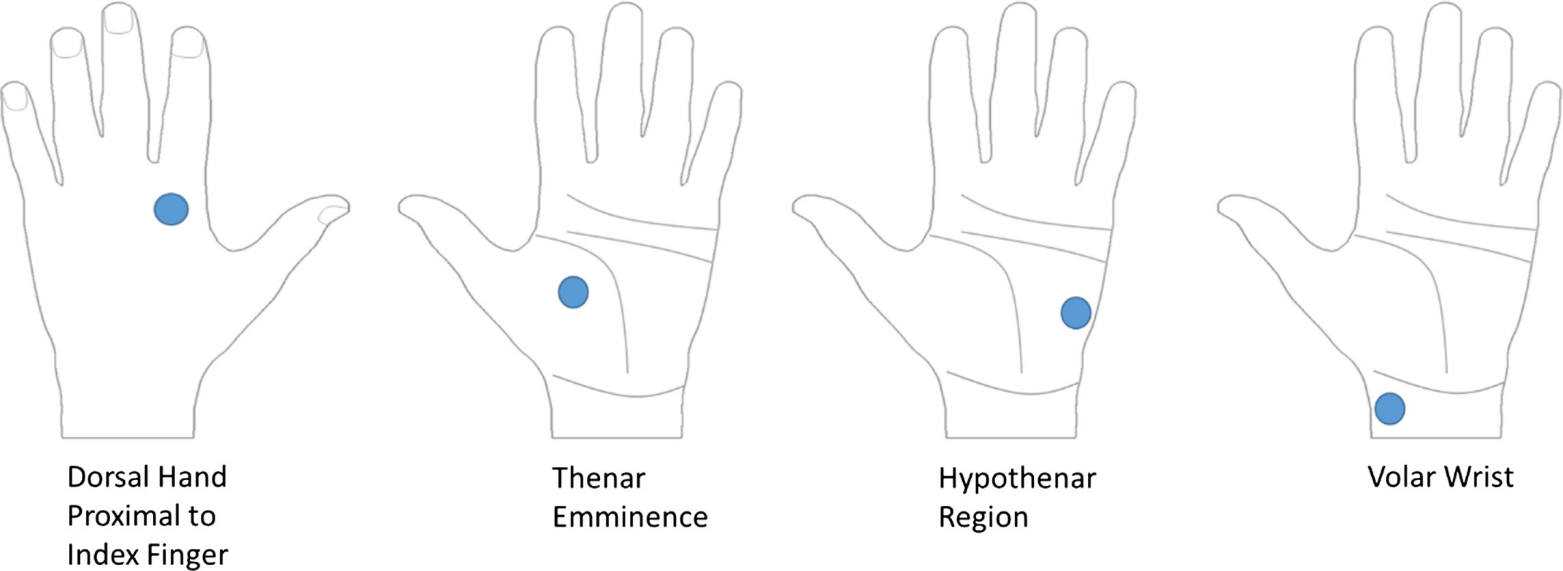
Thumb and index fingertip tactile sensation scores were recorded while white-noise vibration was applied to one of four remote locations: (1) dorsum of the hand just proximal to the second knuckle; (2) thenar eminence; (3) hypothenar region; and (4) volar wrist.

Subjects’ tactile sensation for the thumb and index fingertips was compared with versus without remote white-noise vibration at three vibration intensities (60%, 80%, and 120% of the sensory threshold) and four remote vibration locations (dorsal hand just proximal to the second knuckle, thenar eminence, hypothenar region, and volar wrist as shown in Figure1). The nondominant hand was used because the nondominant hand is thought to be more sensitive to somatosensory feedback than the dominant hand (Haaland and Harrington 1996; Bagesteiro and Sainburg 2003; Sainburg and Schaefer 2004).

The remote vibration was applied by attaching a C-3 Tactor (Engineering Acoustics, Inc. Casselberry, FL) on one of the four locations using tape. The C-3 Tactor generated white-noise vibration low pass filtered at 500 Hz. The remote vibration intensity was adjusted to zero (no vibration), 60%, 80%, or 120% of the sensory thresholds of each remote location. The sensory threshold was the minimal vibration intensity that could be felt by the subject. The sensory threshold was determined by incrementally increasing or decreasing the voltage input to the vibrator (vibration intensity) repeatedly until the subject was barely able to distinguish vibration on versus off as in the method of ascending and descending limits (Collins et al. 1997; Ehrenstein and Ehrenstein 1999). Subjects were verbally asked if they can distinguish vibration on versus off. The average root mean square vibration intensity for the sensory threshold was 0.41, 0.35, 0.37, and 0.55 V for the dorsal hand just proximal to the second knuckle, thenar eminence, hypothenar region, and volar wrist, respectively. Voltage input linearly changes the peak-to-peak vibration displacement, and the average remote vibration at 0.42 V corresponds to peak-to-peak vibration displacement of 0.08 mm according to the manufacturer datasheet.

**Figure 2:**
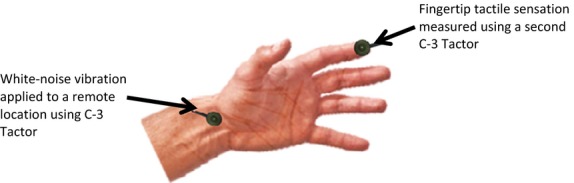
One vibrator (C-3 Tactor) was placed on a remote location (volar wrist in this figure) to provide white-noise suprathreshold (perceivable) or subthreshold (imperceptible) vibration, while a second vibrator was placed on the fingertip pad (the index fingertip in this figure) to measure the fingertip tactile sensation score.

Simultaneously with the remote vibration, another C-3 Tactor was attached to either the thumb or index fingertip to measure fingertip tactile sensation (Fig.2). The thumb and index fingertip tactile sensation score was quantified as the minimum root mean square voltage (V) driving the C-3 Tactor whose stimuli could be barely felt by the subject. The same sensory threshold determination method as described above for the remote vibration was used to determine the fingertip tactile sensation score. During measurement of the fingertip tactile sensation score, the remote vibration was continuously on, whereas the vibration to the fingertip was turned on and off frequently to ask subjects whether they could feel the fingertip vibration or not. A smaller fingertip tactile sensation score indicates better sensation.

The testing order of the four remote vibration locations was randomized. Within each location, the testing order of the vibration intensities (zero, 60%, 80%, and 120%) and fingers (thumb and index) was randomized. The testing session for each subject lasted for approximately two hours.

### Data analysis

Repeated measures analysis of variance (ANOVA) was performed to determine if the fingertip tactile sensation score varied significantly with remote white-noise vibration. An inverse transformation was applied to the fingertip tactile sensation score data to ensure normality (Tabachnick and Fidell 2007). Analysis of variance was performed on the transformed data with the factors of vibration intensity (zero, 60%, 80%, and 120%), vibration location (dorsal hand just proximal to the second knuckle, thenar eminence, hypothenar region, and volar wrist), finger (thumb and index), and their interactions. A conservative significance level of 0.01 was used. Tukey’s post hoc analysis was performed for pairwise comparisons for significant factors.

## Results

**Figure 3:**
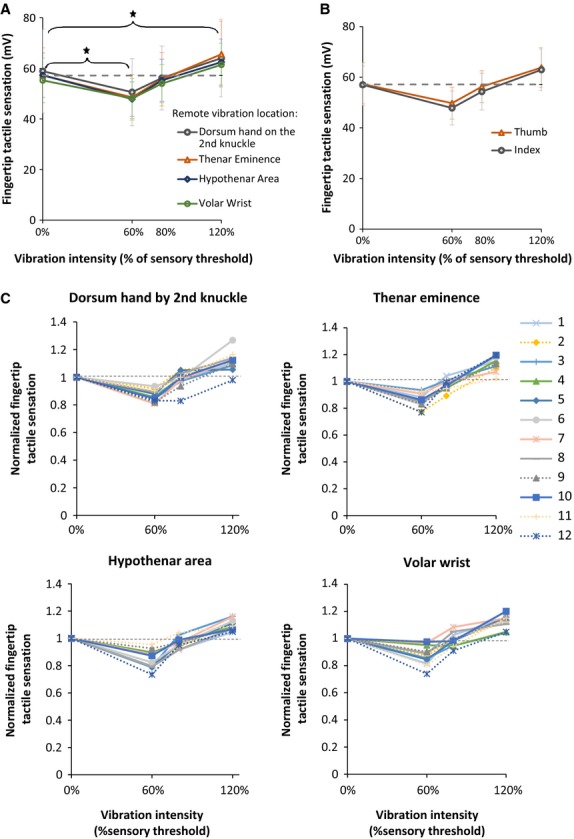
Fingertip tactile sensation scores without and with white-noise vibration at different noise intensities and locations are shown (A). Fingertip tactile sensation scores are fingertip tactile sensory thresholds expressed as the minimum voltage needed to drive the vibrator whose vibration could be detected by the subject’s fingertip pad (minimum perceptible vibration intensity). Fingertip tactile sensation scores changed significantly with intensity of remote white-noise vibration applied to the back of the hand, palm, or wrist (*P < 0.0005) (A). Specifically, the mean fingertip tactile sensation score decreased (improved) with imperceptible remote white-noise vibration at 60% of sensory threshold, compared with no remote vibration (*P = 0.0001). The fingertip tactile sensation score increased (worsened) with perceptible remote white-noise vibration at 120% of sensory threshold, compared with no remote vibration (*P = 0.0072). The 80% white-noise vibration intensity at one of the four remote vibration locations did not significantly change the fingertip tactile sensation (*P* = 0.7667). Averaged data across the fingers and subjects are shown with the stars indicating significant differences from post hoc results (A). This effect of white-noise vibration intensity was observed for all four remote vibration locations (A) for both fingertips’ sensation scores (B). All error bars indicate confidence intervals. In addition to the group data (A), individual subjects’ data are shown for each remote white-noise vibration location, with fingertip tactile sensation scores normalized to each individual’s baseline score (with zero vibration), and the two fingers averaged (C).

The fingertip tactile sensation score significantly varied with vibration intensity (Fig.3, *F*_3,341_^ ^= 79.11, *P *<* *0.0005 in ANOVA). All other effects of vibration location (*F*_3,341_ = 1.30, *P *=* *0.273), finger (*F*_1,341_ = 1.07, *P *= 0.301), and interactions were not found to be significant. Specifically for the effect of vibration intensity, the mean fingertip sensation improved by 15% with vibration at 60% of the sensory threshold compared to no vibration (Fig.3A, T_341_ = −4.335, *P = *0.0001 in post hoc). The vibration intensity of 80% did not result in a significant change in fingertip tactile sensation compared to no vibration (T_341_ = −0.970, *P = *0.7667 in post hoc). Fingertip tactile sensation degraded by 11% with 120% vibration intensity compared to no vibration (T_341_ = 3.213, *P = *0.0072 in post hoc). Such an effect of the vibration intensity was observed for all vibration locations (Fig.3A, C, *F*_9,341_ = 0.16, *P = *0.997 for the interaction between vibration intensity and vibration location in ANOVA). Also, the effect of the vibration intensity was observed for both fingertips (Fig.3B, *F*_3,341_^ ^= 0.16, *P *=* *0.925 for the interaction between vibration intensity and finger in ANOVA). Individual subjects’ data are also shown in Fig.3C. All subjects showed improved fingertip sensation with vibration at 60% of the sensory threshold compared to no vibration (0%) for all vibration locations. All subjects showed worsened fingertip sensation with vibration at 120% of the sensory threshold compared to no vibration for all vibration locations, except for one subject for dorsum hand proximal to the second knuckle.

## Discussions

### Effect of remote white-noise vibration on fingertip tactile sensation

The main finding of this study is that tactile sensation of the thumb and index fingertip pads was affected by application of white-noise vibration to upper extremity skin sites other than fingertips at both imperceptible and perceptible intensities in healthy adults. Specifically, fingertip tactile sensation improved with remote imperceptible vibrations at the intensity of 60% of the sensory threshold. Imperceptible vibrations with the intensity at 80% of sensory threshold did not significantly change fingertip tactile sensation. Perceptible white-noise vibration at 120% of sensory threshold worsened tactile sensation for both thumb and index fingertip pads. Interestingly, these effects of remote vibration of varying intensities on fingertip tactile sensation were found for all four locations to which the white-noise vibration was applied and for two fingertip pads for which tactile sensation was assessed. The way the thumb and index fingertip tactile sensation is influenced by white-noise vibration at the wrist, base of the palm, and back of the hand is postulated below.

### Potential mechanism of remote white-noise vibration affecting fingertip tactile sensation

At the receptor level, it is postulated that not only perceptible but also imperceptible white-noise vibration activated mechanoreceptors in the skin site to which the remote vibration was applied. The minimum intensity of tactile stimuli on the palm to activate sensory neurons (neuronal threshold) was shown to be lower than the minimum intensity of tactile stimuli on the palm that is perceptible to a person (perceptual threshold) (Vallbo and Johansson 1984). Thus, it is likely that the imperceptible vibration activated the skin mechanoreceptors and sensory afferents innervating the wrist, base of the palm, and back of the hand, while not perceived by the persons (Nierhaus et al. 2015). Since the remote vibration had white-noise low pass filtered at 500 Hz, all four mechanoreceptors could be stimulated, with Pacinian corpuscle likely stimulated the most for its sensitivity to vibration. No definitive evidence exists as to if this weak vibration could reach tendon or muscle and stimulate spindles, although tendon vibration is typically performed with substantially suprathreshold vibration intensity with a vibrator pushed into the skin overlaying the tendon unlike the preparation used here with the vibrator lightly placed on the skin.

Similar effects of all four remote vibration locations on fingertip sensation suggest that vibration affects fingertip sensing centrally, as opposed to peripherally. Specifically, the likelihood that the vibration may have traveled along the skin over the 10–20 cm distance to reach and affect the fingertip’s mechanoreceptors is slim, given that vibration loses approximately 90% of its power as it travels 1 to 2 cm along the skin due to the skin’s viscoelastic properties (Manfredi et al. 2012). Also, the effect of vibration found in the results of this study was not related to the distance between the vibration location and the fingertips. Furthermore, vibration is unlikely to have led to direct mechano-electrical stimulation of the median nerve (responsible for fingertip sensation), since stimulation of the dorsum hand or hypothenar area, not overlapping the median nerve, led to the same result. Direct facilitation of the median nerve through action potential propagation within the nerve is also unlikely, since only one vibration location (thenar eminence) shared the median nerve with the fingertips and the other three vibration locations did not involve the median nerve in their pathways.

Centrally, neuronal activity induced by the remote white-noise vibration at the wrist, palm, and back of the hand could influence fingertip tactile sensing through the complex dynamics of the brain. Specifically, application of low-level noise to a neural system has been shown to increase phase synchronization between brain areas assessed by techniques such as the EEG (Ward 2009; Ward et al. 2010). Facilitation in neural synchronization is indicative of enhanced transient communication networks for perception (Ward et al. 2010). As such, when application of low-level sensory noise to one body part facilitates neural synchronization in the brain, another body part’s ability to detect sensory signals can improve: Visual noise to one eye or auditory noise to one ear led to phase synchronization of EEG signals among brain areas and enhanced signal detection with the other eye (Mori and Kai 2002; Kitajo et al. 2003, 2007) or with the other ear (Ward et al. 2010). In addition, crossmodal effects such as enhanced finger tactile and visual sensory threshold with auditory noise was also reported, potentially via the same noise-induced neural synchronization mechanism representative of establishment of transient networks for improved perception (Lugo et al. 2008). Likewise, the remote tactile noise-induced changes in fingertip tactile sensation shown in the present study along with the previous studies (Enders et al. 2013; Hur et al. 2014; Wang et al. 2015) may have been mediated by noise-induced changes in neural synchronization involving brain networks for sensory perception.

As for the effect of white-noise intensity, remote white-noise vibration affected fingertip tactile sensation in a manner similar to stochastic resonance: Low-level imperceptible noise improved tactile signal detection, while perceptible (suprathreshold) noise degraded tactile signal detection (Collins et al. 1997; Wells et al. 2005). Optimal noise intensity of 60% of the sensory threshold, less effective intensity of 80%, and degrading intensity of 120% found in this study coincide with optimal noise range, less effective noise range, and degrading noise range, respectively, from previous studies (Collins et al. 1997; Wells et al. 2005), although the present study delivered the noise and signal to two different skin sites within the upper extremity as opposed to a single skin site as in the previous studies. The postulated involvement of neural synchronization is not contradicted by the noise intensity effect: In fact, it was found that there was an optimal noise intensity that facilitated neural synchronization, whereas too high a noise intensity disrupted neural synchronization (i.e., stochastic resonance in neural synchronization) (Ward 2009). Thus, white-noise vibration at 60% of the sensory threshold at the remote locations may have facilitated neural synchronization, whereas 120% disrupted neural synchronization in the present study. Perceptible vibration at remote locations could also have reduced available attentional resources for fingertips.

In summary, it appears that the remote white-noise vibration may have affected neural synchronization for perceptual sensing thereby changing fingertip tactile sensation, although direct electrophysiologic evidence is warranted to test this viewpoint. In that respect, the neurobiological basis of this sensory manipulation method using remote sensory noise appears to be different from sensory noise applied directly to the fingertip that affects thresholds at the mechanoreceptor level (Collins et al. 1996, 1997; Liu et al. 2002; Kurita et al. 2013). In addition, the neurobiological basis of this sensory manipulation method appears to be different from the coactivation paradigm in which the two-point discrimination threshold of a fingertip pad improves after the fingertip pad receives suprathreshold vibration for 20 min or 3 h, potentially via synaptic plasticity induced by coactivation of neighboring mechanoreceptors within the fingertip pad (Dinse et al. 2006; Dinse and Tegenthoff 2015).

### Practical implications

This study suggests a new sensory manipulation paradigm for fingertip tactile sensation using white-noise vibration applied to different skin areas in the distal upper extremity. Specifically, imperceptible or perceptible white-noise vibration could be used to improve or degrade fingertip tactile sensory threshold, respectively, depending on particular applications. For instance, since finger tactile sensation is essential for dexterous hand function (Zatsiorsky and Latash 2004), imperceptible white-noise vibration at the wrist or other parts of the upper extremity may be used to facilitate hand dexterity for high-precision manual dexterity tasks (Kurita et al. 2013) or to improve hand function for those with finger sensory deficit and subsequent hand impairment (Seo et al. 2014). Such fingertip sensory manipulation is achievable with a relatively low-cost vibrator generating low-risk small vibration. In addition, the vibration does not have to be applied directly to the tactile signal source, but rather to a distant skin site such as the wrist or the back of the hand, which offers an advantage of placing a vibrator away from the fingers so as not to physically interfere with finger movement, finger sensing, and function.

Only tactile sensory threshold for vibratory stimuli was examined in this study. While improved performance on the two-point discrimination and texture discrimination tests was observed with imperceptible white-noise vibration applied to the side of the fingertip in healthy adults (Kurita et al. 2013), such an effect lacked for the two-point discrimination test in chronic stroke patients with white-noise vibration applied to other upper extremity sites (Enders et al. 2013). The way remote white-noise vibration affects other aspects of tactile sensation such as discrimination and resolution in healthy adults needs to be further investigated.

## Conclusions

Remote white-noise vibration affected perceptual detection of fingertip tactile signal in healthy adults. Specifically, white-noise vibration at the intensity of 60% of sensory threshold improved fingertip tactile sensation, while vibration at 120% of sensory threshold degraded fingertip tactile sensation. This effect of remote white-noise vibration was found for all four remote locations in the hand and wrist to which the vibration was applied as well as for the two fingertips for which tactile sensation was measured. These results suggest that remote white-noise vibration exhibits stochastic resonance-type behavior in affecting fingertip tactile sensation.

## Conflict of Interest

Its contents are solely the responsibility of the authors and do not necessarily represent the official views of the NIH. Seo is an inventor of a pending patent using low-level vibration for sensory enhancement.
